# Challenges faced by health volunteers in comprehensive health centers in the southwest of Iran: A qualitative content analysis

**Published:** 2018

**Authors:** Fatemeh Vizeshfar, Marzieh Momennasab, Shahrzad Yektatalab, Mohamad Taghi Iman

**Affiliations:** * Department of nursing, School of nursing and midwifery, Shiraz University of Medical Sciences, Shiraz, Iran; ** Department of Nursing, School of Nursing and Midwifery, Shiraz University of Medical Sciences, Shiraz, Iran; *** Faculty of Nursing & Midwifery Department of Mental Health & Psychiatric Nursing Shiraz University of Medical Sciences Shiraz,Iran; **** College of Social Sciences, Department of Sociology, Shiraz University Shiraz, Iran

**Keywords:** volunteers, Health, Content analysis, Qualitative research

## Abstract

**Introduction:**Health volunteers are employed to execute governmental health promotion programs in most countries around the world. The present study aimed to assess the challenges of health volunteers program to provide a better understanding of the present situation.

**Methods:**This study was conducted using a qualitative approach. 14 participants were selected purposively selected from two comprehensive health care centers in the southwest of Iran. The data were collected through 14 semi-structured interviews, 2 observations, and 3 diaries and analyzed using content analysis.

**Results:**Data analysis resulted in emergence four themes, namely role confusion, the inefficiency of volunteers training, the inefficiency of attraction and maintenance of volunteers, and being unknown to people. Unfertilized capacity is the main theme extracted from all themes.

**Conclusion:**Health volunteers’ perfect accomplishment of roles will have a positive impact on the provision of primary healthcare services and health objectives. Hence, comprehensive planning based on the needs of health volunteers will change them into a strong arm in the health system. Thus, managerial support and precise planning seem to be necessary for facing these challenges.

## Introduction

In most countries around the world, health volunteers are employed to execute governmental health promotion programs and provide social welfare services. In fact, health volunteers act as the link between the society and the healthcare system in different countries [**1**][**2**][**3**][**4**]. Hence, health volunteers are the valuable resources of all non-profit, governmental, commercial, and humanitarian organizations [**5**]. Many studies have also referred to the health volunteers’ positive roles in the execution of health programs. For instance, mother and child services and fighting against malaria, brucellosis, etc. have been done by health volunteers in different parts of the world [**6**][**7**][**8**]. Moreover, health volunteers play a significant role in the improvement of people’s experience of care, create a strong relationship between the society and services, lead to the integration of care services, improve general health, and reduce inequity in health [**9**]. In Iran, health volunteers are selected from women residing in different urban regions. In addition to governmental employees, these women play their roles in the improvement of the society’s health without receiving income [**10**]. Unfortunately, a limited number of studies have been conducted on the attraction and maintenance of health volunteers and the reason why they quit their job suddenly [**11**]. However, evidence has indicated that various factors, including personal, social, and health system-related factors, affect such individuals’ motivation and performance. Personal factors include humanitarian viewpoint towards others and society, fulfilling one’s dreams, acquiring skills, and hope to receive financial rewards or get employed in the health system. On the other hand, the weak adaptation of personal goals to organizational ones results in burnout and tendency towards quitting the job [**12**]. Social factors include being recognized and accepted by others, familial support, and financial and job-related support. Finally, the factors related to the health system include instruments and facilities, training, supervision, and job description. In this respect, the challenges faced by health volunteers consist of insufficient financial rewards, high workload, insufficiency of services for the society, insufficient supervision, and insufficient support services such as transportation. In Iran, health volunteers are encountered with challenges that lead to their separation from the health system as well. These challenges include people’s lack of familiarity with health volunteers program, lack of welfare and motivational facilities and inattention to volunteers’ suggestions and requirements. On the other hand, familial support, appropriate relationship with instructors and others, and the existing social network have been mentioned as the effective factors in the promotion of health volunteers’ cooperation [**10**][**13**]. However, few studies have been conducted in this regard in Iran. Besides, most of the studies have been designed quantitatively and evaluated the impacts of health volunteers’ cooperation in various health programs [**10**][**13**][**14**][**15**]. Considering the effects of cultural and social factors on health volunteers and multi-dimensionality of the effective factors in services, qualitative researches can objectively reveal the realities since they focus on individuals’ opinions, experiences, and emotions. Therefore, using such studies, concepts not emphasized in quantitative researches can be extracted from the existing realities. A qualitative approach to healthcare can also help explore the phenomena faced by nurses, other decision-makers, and patients, eventually resulting in a deep understanding of the issues [**16**][**17**][**18**]. Content analysis is one of the qualitative research methods and a suitable method for deep access to human experiences. In this method, realities are interpreted based on the systematic categorization of scientific data and mental interpretation of textual information content. This can be used as a guideline in order to reach a new insight [**19**][**20**]. Despite the important role of health volunteers in the health system, it seems that sufficient attention has not been paid to planning for attraction and maintenance of these individuals and elimination of their problems. This can lay the ground for exclusion of health volunteers from the program or inappropriate utilization of their capacities. Hence, the present study aims to determine the challenges in Iran health volunteers program and provide an opportunity for better understanding of its status.

## Methods

**Ethics**

This study was approved by the Ethics Committee of Shiraz University of Medical Sciences, Shiraz, Iran (No. IR.sums.REC.1394.112). All the participants were informed about the study objectives and signed written informed consent for taking part in the study. They were also reassured that not participating in the study had no effects on their status. Besides, they were free to leave the study at any time. Moreover, the participants’ privacy and confidentiality were observed all through the study period.

This qualitative study was conducted in two health centers in the southwest of Iran in Shiraz, which covered almost 5000 and 3000 households, respectively. It should be noted that urban health centers in Iran provide first and second level preventive health services. After obtaining written informed consent, with purposive sampling, 14 health volunteers were enrolled into the study. The inclusion criteria of the study were being willing to take part in interviews, being accessible and having participated in the health volunteers program for at least 6 months. The study data were collected through 14 semi-structured interviews in Persian, 2 observations, investigation of 3 diaries, and evaluation of the volunteers’ records by the first author from September 2015 to March 2016. It should be noted that the observations were focused on the volunteers’ performances and problems. Additionally, 3 health volunteers were required to provide their diaries regarding the activities they carried out throughout a day. Moreover, the health volunteers’ records were examined for their demographic information and reports of their performances. Data collection was continued until reaching data saturation. In order to direct the interviews, a guideline including several key questions was prepared. Accordingly, the interviews were begun with the following statement: “Describe your workday”. Then, based on the interviewees’ responses, the following questions were proposed: “How do you think you will have a better performance” and “What are the weak points of the health volunteers program”. Probing questions, such as “Explain more”, were also used. Each interview lasted for 40-60 minutes.

**Data analysis**

Inductive and conventional content analyses were used to explore the health volunteers’ challenges. Data collection and analysis were done simultaneously. At first, the interviews were recorded with the volunteers’ permission, transcribed on paper, and coded. Also, information about observations and daily notes were coded as well, and finally data has been merged using triangulation. Primary coding was performed based on the words utilized by the volunteers and the researcher’s inductions. Then, meaning units were extracted from the data in the form of primary or open codes. After that, the codes were reviewed for several times and were classified based on their similarities. Finally, the categories were compared, similar ones were merged, and the main themes were determined.

**Trustworthiness**

The accuracy of each research paper is approved by the trustworthiness of the data. In this study, Guba and Lincoln’s criteria, including credibility, transferability, dependability, and conformability were used to reassure the accuracy of the research [20, 21]. Due to this, various methods were employed through the study in order to ascertain data accuracy. For instance, credibility was determined by reviewing the codes and categories by the health volunteers, prolonged engagement, researcher’s and volunteers’ agreement on the codes, and neutrality. Using various data collection methods and triangulation also helped determine data accuracy.

## Results

All the study health volunteers were female and married. Their ages ranged from 38 to 70 years, with the mean age of 49.8+8.65 years. Besides, their job tenure ranged from 3 to 15 years, with averaging 10.7+4 years. Moreover, one health volunteer (7.1%) had an associate's degree, while the remaining 13 (92.8%) had high school and diploma degrees. Overall, one main theme, 4 themes and 14 subthemes were extracted from the data. The main theme and subthemes of the health volunteers’ challenges are shown in **Table 1**.

**Table 1:** The main theme and the related subthemes emerged from the data

Main theme	Themes	Subthemes
Unfertilized capacity	Role confusion	Limited participation in various roles
		Lack of specific planning for health volunteers' activities
		Lack of comprehensive orientation programs
		Lack of knowledge about responsibilities
	Inefficiency of volunteers training	Small number of training programs
		Doing affairs without basic trainings
		Lack of educational facilities
	Inefficiency of attraction and maintenance of volunteers	Lack of appropriate programs for attracting health volunteers
		Insufficient support and supervision
		Weakness in volunteers' and instructors' communication skills
		Lack of feedback to health volunteers' activities
	Being unknown to people	People's unfamiliarity with health volunteers' names
		People's unfamiliarity with health volunteers' activities
		People's lack of interest in cooperating with health volunteers

**1- Role confusion**

The first theme extracted from the data was the health volunteers’ role confusion, which included the following subthemes: limited participation in various roles, lack of specific planning for health volunteers’ activities, lack of comprehensive orientation programs, and lack of knowledge about responsibilities. In this study, most of the participants had a sort of role confusion, such a way that they did not know their responsibilities and did not have specific programs. All the participants mentioned the lack of a specific program for their activities as a problem. Besides, they stated that they trained mostly their family members, friends, and neighbors accidentally without any particular programs: “I say what I have learned to my husband and children. I also tell my neighbors when I see them or when they ask” (a 36-year-old woman with 1.5 years of experience as a health volunteer).

Moreover, the health volunteers’ responsibilities were not clarified and they did not have any integrated programs. In fact, they just had some scattered information about the goals of the health volunteers program. Also, their participation in service provision, such as screening examination, did not follow any specific training or proper planning: “Our instructor hasn’t told us anything about training people. Some (health volunteers) go to school with her and help her” (a 47-year-old woman with 5 years of experience as a health volunteer).

**2- Inefficiency of volunteers training**

Another theme extracted from the study data was the inefficiency of volunteers training with the following subthemes: a small number of training programs, doing affairs without basic training, and lack of educational facilities. The health volunteers believed that the educational content they received as the official educational program did not meet their educational needs. In fact, the educational content was mostly theoretical and limited time was contributed to skills training. Besides, the theoretical issues were mostly presented through lecture rather than learner-oriented methods: “Now, the health volunteers program is limited to theoretical training. For example, we aren’t trained about skills such as blood pressure measurement” (a 48-year-old woman with 15 years of experience as a health volunteer). “First, contents are repeated. I became a health volunteer when my first daughter was 1-2 years old. Now, she is 14. I have been reviewing these books for 12-13 years. It is repeated, believe me. Sometimes, I’m not even interested in listening to what is said in the class. I know it all” (a 37-year-old woman with 12 years of experience as a health volunteer).

Another problem mentioned by the health volunteers was related to the lack of educational aids. There were limited numbers of educational facilities, such as books and pamphlets, to help the health volunteers remember and apply what they had learned: “Educational subjects were given to us in the form of pamphlets in the past. These are completely omitted now. We don’t have them. When we entered the program, it was good; they gave us the educational content in the form of pamphlets. We still have some of them now but unfortunately, all these are gone now; they don’t give us the educational contents in writing” (a 46-year-old woman with 15 years of experience as a health volunteer).

Observation 1: “The instructor has a book in her hand and explains about brucellosis based on what is written in the book. However, most of the health volunteers just listen. After the end of the class, the instructor expressed a summary, but no pamphlets were given to the volunteers”.

**3- Inefficiency of attraction and maintenance of health volunteers**

This theme consisted of the following subthemes: the lack of appropriate programs for attracting health volunteers, insufficient support and supervision, weakness in volunteers’ and instructors’ communication skills, and the lack of feedback to health volunteers’ activities. Most of the health volunteers stated that they had gotten familiar with the program quite accidentally. Besides, there were limited motivational programs for the health volunteers and nothing special was done in practice. They also believed that no sufficient attempts were made to attract and maintain the health volunteers. Moreover, evaluation of the health volunteers’ performance was based on their own or their instructors’ reports, and no significant difference was there between active and inactive health volunteers. Furthermore, there were limited motivational programs, such as going camping, compared to the past. Overall, the health volunteers were attracted to the program by other health volunteers, and no specific program was there in this regard. One of the participants maintained: “We were out with friends and they suddenly said that there were some meetings in the clinic. They didn’t say they were volunteers. They said there were meetings in the health center where we learn good materials about diseases. So, we came as well” (a 45-year-old woman with 9 years of experience as a health volunteer).

“It would have been better if there were more recreational programs, more facilities. Women contribute their time, but there’s nothing encouraging” (a 50-year-old woman with 12 years of experience as a health volunteer).

“I don’t want anything. It’s just for God’s sake. But they’d better encourage the women, give them rewards, for example, because there are few rewards. Or they could celebrate International Volunteer Day because women come and feel very happy but these aren’t enough” (a 45-year-old woman with 9 years of experience as a health volunteer).

**4- Being unknown to people**

Another theme obtained in this study was people’s unfamiliarity with the health volunteers, which could have a negative impact on their activities. The subthemes of this theme included people’s unfamiliarity with health volunteers’ names, people’s unfamiliarity with health volunteers’ activities, and people’s lack of interest in cooperating with health volunteers. The health volunteers believed that people were not familiar with their names and activities, while they were assumed to serve as the link between people and the health system. People’s unfamiliarity with the health volunteers was quite obvious while observing the volunteers’ performances. Accordingly, since people were not familiar with the health volunteers’ names and responsibilities, they did not cooperate with them in the case of referral to their houses or service provision.

Observation 2: “A blood pressure and BMI screening program was held with the help of the physician in the clinic. Among the 30 participants, only one had information about health volunteers, because she was a health volunteer in the past. The health volunteers did not introduce themselves”.

“When I say I’m a health volunteer, they get surprised. They ask what does it mean, what do I do and I explain to them. People don’t know, most of them don’t know” (a 40-year-old woman with 6 years of experience as a health volunteer).

“Because I explain and train my family, they’ve got used to the health volunteers program. They sometimes joke and say their mother is a doctor. But this is not the case for society as well, they know nothing” (a 45-year-old woman with 9 years of experience as a health volunteer).

**Unfertilized capacity**

Considering the extracted themes, the health volunteers were interested individuals who had the potential to provide the society with healthcare services. However, they were not assisted in extending their potentials. In other words, the health volunteers represented the society members’ cooperation in the improvement of society’s health. Nevertheless, neither the society is aware of them nor do the related authorities pay attention to the improvement of their abilities. Thus, the required ground has not been provided for their service provision. In fact, health volunteers are inclined to help, but they do not exactly know what to do and, consequently, cannot carry out their responsibilities effectively.

“If there are facilities, they can allocate some space in public places to be used by 1-2 health volunteers and instructors to explain to people. That would be great” (a 45-year-old woman with 12 years of experience as a health volunteer).

“I’m interested, but I’m not free. I’ve cooked lunch so that my family won’t complain and then I came here. I go wherever I can learn something, but if it were better, more orderly, I think we would learn and help people more” (a 40-year-old woman with 6 years of experience as a health volunteer).

## Discussion

The present study aimed to determine the challenges faced by health volunteers in Iran to provide an opportunity for better understanding and improvement of their performance. The results indicated that health volunteers were encountered with several challenges in service provision and linking people to the health system. Accordingly, health volunteers had unfertilized capacities that, if taken into account, could improve their performance. This resulted from role confusion, the inefficiency of volunteers training, the inefficiency of attraction and maintenance of volunteers, and being unknown to people. Based on the results, the health volunteers were confused in playing their roles, because they had no specific planning for training people or providing them with services. Also, they received no specific training in this regard in the health centers. Therefore, health volunteers have to cooperate more with the staff while their responsibilities, as well as patients’ and authorities’ expectations, have to be clarified [**22**]. The results of a study conducted in the west of the U.S. revealed that health volunteers experienced role confusion and great stress while taking care of end-stage patients in hospice care because they did not know what they had to do in most cases [**23**].

Role confusion and its resultant stress impose a great pressure on health volunteers. The results of studies on health volunteers’ awareness and performance of their roles indicated that many health volunteers experienced burnout due to problems and pressures [**24**]. Overall, the roles of health volunteers have to be clarified for themselves and other professionals. In this way, the health volunteers’ efficiency will be improved and integrated, and holistic care will be provided [**4**][**8**][**9**]. It seems that making health volunteers aware of their roles and authorities’ expectations would eliminate their confusion, eventually resulting in volunteers, people and authorities’ satisfaction. Another challenge that emerged in the current study was the inefficiency of volunteers training. The health volunteers’ educational programs included several books developed based on primary healthcare provision, which were taught through lecture one hour a week. Nonetheless, wider training related to particular problems of different regions and learning of skills are required for better performance. Additionally, using learner-centered teaching methods and appropriate educational aids would lead to better learning. Overall, good skills and practice are necessary for health volunteers [**9**]. A large number of health volunteers expressed the necessity of planners’ and managers’ attention in order to obtain essential skills [**4**]. Considering the ever-changing health requirements in different groups and societies, health volunteers need to receive up-to-date trainings and acquire proper skills. Increasing knowledge and skills would, in turn, empower health volunteers and enhance their confidence [**25**][**26**]. Effective training of health volunteers is also necessary for effective changes in their programs [**26**]. Ploeg et al. assessed 16 health volunteers’ tendency towards facilitating personalized activities for patients with Alzheimer’s disease in Australia. In spite of problems in looking after such patients, the health volunteers were satisfied with gaining new knowledge and skills [**25**]. Overall, it seems that the more the health volunteers learn and feel beneficial, the higher their motivation is [**24**]. Another challenge in this study was related to the health volunteers’ attraction and maintenance. In fact, attraction and employment of the health volunteers was a major challenge for them and the health system. According to the study results, no appropriate programs were there for attraction of the health volunteers. Besides, they believed that they did not receive the necessary support, supervision, and encouraging feedback and they had a weak relationship with their instructors. Generally, health volunteers’ main challenges were support, correct management, reassurance about the quality of their performance, and their relationship with other staff members in the organization [**9**]. In Iran, a study was carried out to determine the effective factors in women’s tendency for being healthy volunteers. The results of that research indicated a significant relationship between tendency to volunteer in the health field and the supporting social network, including family, society, and healthcare system [**10**]. Investigation of health volunteers taking care of individuals at end stages of life in England showed different challenges, which indicated the necessity to change this program for effective performance of health volunteers. The most important challenges were related to the attraction and maintenance of health volunteers and existence of support and supervision structures. Moreover, some individuals emphasized the effectiveness of rewards and feeling beneficent as a part of the required changes [**26**]. Furthermore, planners and instructors play a critical role in the maintenance of health volunteers. In fact, planners play the vital role in attracting and maintaining health volunteers for service provision and have to evaluate their needs and support them. In many cases, inattention to health volunteers’ needs and improvement resulted in disruption of their cooperation with the health centers [**13**]. However, support and supervision were among the factors leading to health volunteers’ success and satisfaction [**25**]. Nonetheless, not employing proper mechanisms for attracting and maintaining health volunteers would result in disorders in the system that is going to use them for health services provision, which can eventually have negative effects on the quality and quantity of services [**3**][**10**][**27**]. Therefore, further studies have to be conducted on the attraction and maintenance of health volunteers in health systems, the required training, and their socialization [**27**]. Furthermore, appropriate relationships and feedback to the health volunteers would lead to the creation of a comprehensive communication network and enhance health volunteers’ tendency to cooperate [**24**]. On the other hand, weak relationships and low motivation are among the main barriers against service provision by health volunteers [**6**]. Another theme extracted from the current study was being unknown to people, which caused people not to cooperate with the health volunteers. Managerial support might also be effective in this regard. However, this problem was not reported in other parts of the world, which might be due to differences in place and method of providing services. A study in China assessed elderly individuals’ demand for receiving services for chronic diseases from health volunteers. Based on the results, most elderly individuals were interested in the health volunteers program. Thus, the health and treatment system was recommended to support service provision by health volunteers and provide them with proper and sufficient training in order to improve their services’ standards [**28**]. The first step in providing services is the familiarization of families and services consumers, which results in a proper relationship and clients’ readiness for receiving services [**22**]. Besides, familiarity and trust are the prerequisites for service provision without which provision and reception of services would be disrupted significantly. This should be carried out by the organization using health volunteers for providing services [**13**][**26**][**29**]. The main theme of the current study was unfertilized capacity. This implied that in spite of the health volunteers’ potential capacities, the health system has not managed to benefit from their potentiality. In general, health volunteers start their career because of their interest in learning and helping improve the society’s health, which is considered to be a strong point for the health system. Additionally, the lack of specific roles, insufficient sources for health volunteers’ training and service provision, the lack of systematic structures and authorities’ support, the lack of comprehensive planning, and devaluing health volunteers caused them not to use their capacities completely for providing services [**22**]. Health volunteers also believed that they needed to acquire various skills to carry out their responsibilities perfectly. They also had to understand well and be satisfied with their roles [**30**]. Positive feedback, staff’s support, and health volunteers’ learning also encouraged them to continue their cooperation. Therefore, measures should be taken to eliminate health volunteers’ shortcomings considering their location of service provision [**31**]. Thus, the society has to be informed in this regard. Besides, the health volunteers program has to be reviewed and modified based on the changes in society’s needs, health system’s plans, and health volunteers’ problems in order to improve the society’s health [**13**].

## Conclusion

Despite successes, the health volunteers program is facing several challenges. These challenges can be eliminated by clarifying the roles of health volunteers through comprehensive planning based on their educational needs, supporting health volunteers to gain the necessary knowledge and skills for providing society with training and services, developing measures for the attraction and maintenance of health volunteers, making people familiar with the services they provide, and further utilization of health volunteers’ services by providing them with the required support resources. These can eventually empower the health volunteers to supply the society with beneficial, efficient, and cost-effective services. In fact, supporting this program will increase the society’s responsibility towards its members’ health. The findings of the present study revealed the necessity to develop new strategies that can improve volunteers’ performances, which can, in turn, facilitate changes for increasing their satisfaction. These can lead to a higher level of community health promotion. Future research should be conducted on addressing health volunteers’ challenges. Therefore, identified problems have to be addressed by the health volunteers who are a part of the process to act on their behalf to solve real-world problems. Overall, health volunteers’ perfect accomplishment of roles will have a positive impact on the provision of primary healthcare services and health goals. Hence, comprehensive planning based on health volunteers’ needs will change them into a strong arm in the health system.

**Recommendations**

The results of this study can be used by planners of health volunteers’ services in the health sector in order to identify the weak points of the health volunteers program. Health managers can also utilize them for providing managerial support and proper planning to face these challenges. Additionally, interviewing the consumers of services can enlighten other dimensions of the challenges. Moreover, a needs analysis regarding the shortcomings of this program should be done on people, health volunteers, and cooperating staff in order to complete the results of this research.

**Acknowledgment**

Hereby, the authors would like to express their gratitude to the healthcare volunteers for participating in this study. They would also like to appreciate Dr.Tahamtan, head of the Unknown Martyrs Health Center, and all staff in these health centers for their cooperation.

**Source of funding:**

This article was extracted from a PhD dissertation in nursing education, which was financially supported by the Research Vice-chancellor of Shiraz University of Medical Sciences (grant No. 94-7577).

**Disclosures:** The authors declare that there is no conflict of interest.
